# Inhibition of Receptor Interacting Protein Kinases Attenuates Cardiomyocyte Hypertrophy Induced by Palmitic Acid

**DOI:** 10.1155/2016/1451676

**Published:** 2016-01-04

**Authors:** Mingyue Zhao, Lihui Lu, Song Lei, Hua Chai, Siyuan Wu, Xiaoju Tang, Qinxue Bao, Li Chen, Wenchao Wu, Xiaojing Liu

**Affiliations:** ^1^Laboratory of Cardiovascular Diseases, Regenerative Medicine Research Center, West China Hospital, Sichuan University, Chengdu 610041, China; ^2^Department of Pathology, West China Hospital, Sichuan University, Chengdu 610041, China

## Abstract

Palmitic acid (PA) is known to cause cardiomyocyte dysfunction. Cardiac hypertrophy is one of the important pathological features of PA-induced lipotoxicity, but the mechanism by which PA induces cardiomyocyte hypertrophy is still unclear. Therefore, our study was to test whether necroptosis, a receptor interacting protein kinase 1 and 3 (RIPK1 and RIPK3-) dependent programmed necrosis, was involved in the PA-induced cardiomyocyte hypertrophy. We used the PA-treated primary neonatal rat cardiac myocytes (NCMs) or H9c2 cells to study lipotoxicity. Our results demonstrated that cardiomyocyte hypertrophy was induced by PA treatment, determined by upregulation of hypertrophic marker genes and cell surface area enlargement. Upon PA treatment, the expression of RIPK1 and RIPK3 was increased. Pretreatment with the RIPK1 inhibitor necrostatin-1 (Nec-1), the PA-induced cardiomyocyte hypertrophy, was attenuated. Knockdown of RIPK1 or RIPK3 by siRNA suppressed the PA-induced myocardial hypertrophy. Moreover, a crosstalk between necroptosis and endoplasmic reticulum (ER) stress was observed in PA-treated cardiomyocytes. Inhibition of RIPK1 with Nec-1, phosphorylation level of AKT (Ser473), and mTOR (Ser2481) was significantly reduced in PA-treated cardiomyocytes. In conclusion, RIPKs-dependent necroptosis might be crucial in PA-induced myocardial hypertrophy. Activation of mTOR may mediate the effect of necroptosis in cardiomyocyte hypertrophy induced by PA.

## 1. Introduction

It is well recognized that excessive intake of dietary saturated fatty acids contributes to heart failure [1]. Considering the elevated plasma concentration of free fatty acids (FFAs), this phenomenon is partly explained by the development of obesity, coronary atherosclerosis, and myocardial ischemia [1]. However, dysfunction of cardiomyocytes caused by excessive intracellular lipids accumulation could be a significant other side of this phenomenon. Overload of lipids in nonadipose tissues that affects cellular functions, namely, lipotoxicity, could also induce cell hypertrophy or even cell death [2]. Evidence suggests that accumulation of PA, the major saturated fatty acids in blood, may give rise to lipotoxicity in cardiomyocytes by induction of oxidative stress [3] and persistent ER stress [4].

ER performs a pivotal role in various cell processes, including synthesizing, assembling, modifying and trafficking of proteins, and maintaining intracellular Ca^2+^ homeostasis [5]. Upon ER stress, accumulation of unfolded proteins leads to activation of sensors (PERK, ATF6, and IRE1) via dissociating GRP78 from them and triggers unfolded protein response (UPR). A short-term UPR functions as a prosurvival response via reducing accumulation of unfolded proteins and restoring ER function. If UPR prolongs, its downstream signaling initiates complicated response to strengthen ER stress, activates proapoptosis pathways, and eventually induces cell death [6]. According to previous studies, myocardial hypertrophy and apoptosis induced by PA are accompanied with increased expression of ER stress markers [7].

Moreover, mammalian target of rapamycin (mTOR), which is also essential for cardiomyocyte development, growth, and functions, regulates mitochondrial fatty acid utilization in the heart [8]. The AKT/mTOR signaling pathway has emerged as an important regulator in the pathogenesis of myocardial hypertrophy [9]. In cardiomyocytes, PI3K/mTOR/p70 (S6K) plays a critical role in the leptin-induced hypertrophy [10]. In islet beta-cells, PA activates mRNA translation and increases ER protein load via activation of the mTOR pathway [11]. In adipocytes, inhibition of AKT or mTOR signals by rapamycin attenuates the PA-induced ER stress [10]. However, whether PA evokes ER stress by activating mTOR signaling in cardiomyocytes is unclear, and the underlying mechanism of the PA-induced cardiomyocyte lipotoxicity, more specifically, in the PA-induced cardiomyocyte hypertrophy still remains elusive.

Recent studies have indicated a previously unknown form of programmed necrosis called necroptosis, which is regulated by the RIPK1 and RIPK3. In particular, a kinase complex consisted of the RIPK1 and RIPK3 is a central step in the programmed necrotic cell death [12]. Necroptosis represents a newly identified mechanism of cell death sharing features of both apoptosis and necrosis. Although RIPKs-dependent necroptosis has been implicated in the development of several cardiovascular diseases, such as atherosclerosis [13], myocardial infarction [14], and ischemia-reperfusion injury [15], the potential role of RIPKs-dependent necroptosis in the PA-induced myocardial hypertrophy is still unknown. Therefore, in the perspective of its critical role in inflammation and cell death in cardiovascular diseases, we hypothesized that necroptosis might participate in the pathophysiological process of cardiomyocyte hypertrophy induced by PA.

In order to verify our hypothesis, we sought to examine the influence of PA on the expression of RIPK1 and RIPK3 in NCMs and in H9c2 cells in this study. It has been reported that blockade of mTOR with its specific inhibitor CCI-779 stimulates autophagy and eliminates the activation of RIPKs in RCC4 cells [16]. Accordingly, the crosstalk between necroptosis, ER stress, and AKT/mTOR signaling pathway in cardiomyocytes with PA treatment was also investigated.

## 2. Materials and Methods

### 2.1. Cell Culture and Pharmaceutical Treatments

The NCMs were obtained from decapitated 0 to 3-day-old Sprague-Dawley rats by collagenase II (0.05%) (Gibco) and trypsin (0.05%) digestion according to the methodology of previous studies [17]. The culture medium consisted of DMEM (high glucose) (Gibco) and 10% (v/v) fetal bovine serum (FBS, Hyclone, USA). All cells were maintained in a humidified 5% CO_2_/95% air incubator at 37°C. H9c2, rat embryonic cardiac myoblasts from American Type Culture Collection (ATCC), were grown in DMEM with 10% FBS at 37°C and 5% CO_2_. When cells reached 70–80% confluence, they were incubated with 1% BSA-DMEM with or without PA (200 *μ*M, Sigma) for 24 h. Thapsigargin (100 nM, Sigma) and pravastatin (10 *μ*M, Squibb) were used as the agonist and antagonist for ER stress [18]. Nec-1 (10 nM, Selleck) was a known specific inhibitor for RIPK1, and rapamycin (1 *μ*M, Sigma) was used to block the mTOR signaling activation. All these treatments were pretreated with the NCMs or H9c2 cells for 2 h before the PA stimulation.

### 2.2. siRNA Transfection

Transient transfection was performed by use of the cationic lipid Lipofectamine 3000 (Invitrogen, USA) according to the manufacturer's instruction. The H9c2 cells were transfected for 24 h with 50 nM siRNA specific for RIPK1/RIPK3 or negative control siRNA before exposure to PA (200 *μ*M) for another 24 h. The sequences of siRNAs used were as follows:

 Negative control siRNA (siNC): 5′-GUG CGUUGCUAGUACCAAC dUdU-3′.
 RIPK1-siRNA (siRIPK1/siR1): 5′-GCG GGCAUGCACUACUUACAUG dUdU-3′.
 RIPK3-siRNA (siRIPK3/siR3): 5′-GCG GGGUCAGGAUCGAGAGAUU dUdU-3′.

### 2.3. Real-Time PCR

Gene expression was measured by quantitative RT-PCR (Q-PCR) as our previous report [19]. Total RNA was extracted from cells with TRIzol (Invitrogen, USA), and cDNA was synthesized using a reverse transcription (RT) kit (Toyobo, Japan). Q-PCR was carried out on CFX96 Real-Time PCR Detection System (BIO-RAD, USA) with fluorescence dye SYBR Green (SYBR Green Super mix kit, Bio-Rad, USA). Primer sequences were shown in Table 1. Normalization of gene expression was achieved by comparing the expression of *β*-actin for the corresponding samples. Relative fold expression values were determined applying the ΔΔCT threshold (Ct) method.

### 2.4. Western Blot Analysis

Total proteins were extracted from cells with radio-immunoprecipitation assay (RIPA) lysis buffer. Protein from cell extracts was quantified by Varioskan (Thermo, USA) using a BCA protein assay kit (Pierce, USA). Equivalent amount of cells lysates (25 *μ*g) was separated by denaturing 10% SDS-PAGE and then transferred to 0.45 *μ*m polyvinylidene difluoride (PVDF) membrane (Millipore, USA) using a MiniProtein III system (Bio-Rad, USA). Membranes were subsequently blocked with 5% skim milk in Tris-buffered saline and Tween 20 (TBST) solution for 2 h and were incubated with primary antibodies at 4°C overnight: RIPK3 (Cell Signaling, number 14401), RIPK1 (Cell Signaling, number 3493), GRP78 (Cell Signaling, number 3183), Phospho-mTOR (Ser2481) (Cell Signaling, number 2974), mTOR (Cell Signaling, number 2983), Calreticulin (Cell Signaling, number 12238), AKT (Cell Signaling, number 4685), and Phospho-AKT (Ser473) (Cell Signaling, number 4058). The antigen-antibody complexes were detected by enhanced chemiluminescence (ECL) substrate kit (Thermo, USA). Specific bands were scanned and quantified by the Quantity One analysis software (Bio-Rad, USA).

### 2.5. Detection of Cell Surface Area by F-Actin Staining

Cardiomyocytes surface area was detected by F-actin staining as previously reported [20]. After treatment, H9c2 cells were washed and fixed by 4% paraformaldehyde for 30 min at room temperature. Then the cells were incubated with 0.1% TritonX-100 in PBS for 3 to 5 min and stained with Rhodamine Phalloidin (100 nM, Cytoskeleton) for 20 min. Rinse cells with PBS and incubate them with diluted DAPI for 10 mins, away from light. Images were captured by Eclipse TE2000-U fluorescent microscope system (Nikon, Japan) and semiquantitatively analyzed for cell surface area with ImageJ software (NIH, USA).

### 2.6. Cells Ultrastructure Observation by Transmission Electron Microscopy [21]

After treatment, H9c2 cells were collected and fixed with 3% glutaraldehyde in 100 mM cacodylate buffer, postfixed in 1% cacodylate-buffer osmium tetroxide for 2 h at room temperature, and dehydrated in a graded series of ethanol. Then the cells were embedded in Epon-Aradite. Ultrathin sections were cut with a diamond knife on a Leica EM UC6rt (Leica, German) and double-stained with uranyl acetate and lead citrate. Ultrastructure of H9c2 cells was observed with a Hitachi H7650 transmission electron microscope (TEM, Hitachi, Japan) at 80 kV.

### 2.7. Immunofluorescence Staining

H9c2 cells were plated onto coverslips in 6-well plated. When reaching 60–70% confluent, the cells were treated with PA and Nec-1 or with PA alone. Coverslips were then fixed and blocked as described before [22], followed by exposure to the primary antibodies (anti-RIPK1 1 : 100 or anti-RIPK3 1 : 100, Cell Signaling, USA) at 4°C overnight. After washing with PBS, incubate the cells with fluorescent-conjugated secondary antibodies for 2 h at room temperature, away from light. The second antibody used was Alexa Fluor 488 Goat Anti-Rabbit IgG (1 : 400, green fluorescence, Invitrogen, USA) or Alexa Fluor 594 Goat Anti-Rabbit IgG (1 : 400, red fluorescence, Invitrogen, USA). Rinse cells and incubate them with diluted DAPI for 10 min, away from light. Images were collected using an Eclipse TE2000-U fluorescence microscope system (Nikon, Japan) and analyzed with ImageJ software (NIH, USA) to semiquantitatively determine the expression of RIPK1 and RIPK3.

### 2.8. Oil Red O Staining

To measure intracellular lipid accumulation, H9c2 cells were stained by Oil Red O dye (Sigma-Aldrich, catalog number 398039) according to the methodology of previous study [23]. After treatment, cells were washed and fixed by 4% (v/v) paraformaldehyde. Then the cells were incubated with the Oil Red O working solution for 30 min at room temperature. Subsequently, the cells were washed twice with 60% isopropanol and then counterstained with hematoxylin (Dako, USA) for 30 s. Excess hematoxylin was washed in water. Cells were then observed under the microscope and images were collected using an ECLIPSE 50i system (Nikon, Japan). Oil Red O-staining positive cell counts were determined over five viewing fields and averaged. Totally more than 150 cells in each group were chosen randomly for statistical analysis.

### 2.9. Statistical Analysis

All results were expressed as mean ± SD. We performed statistical analysis using one- or two-way* ANOVA* and Student-Newman-Keuls* post hoc* tests or *t*-tests. A *p* value of <0.05 was considered as significant.

## 3. Results

### 3.1. Cardiomyocyte Hypertrophy Is Induced by Palmitic Acid Stimulation

**Figure 1:**
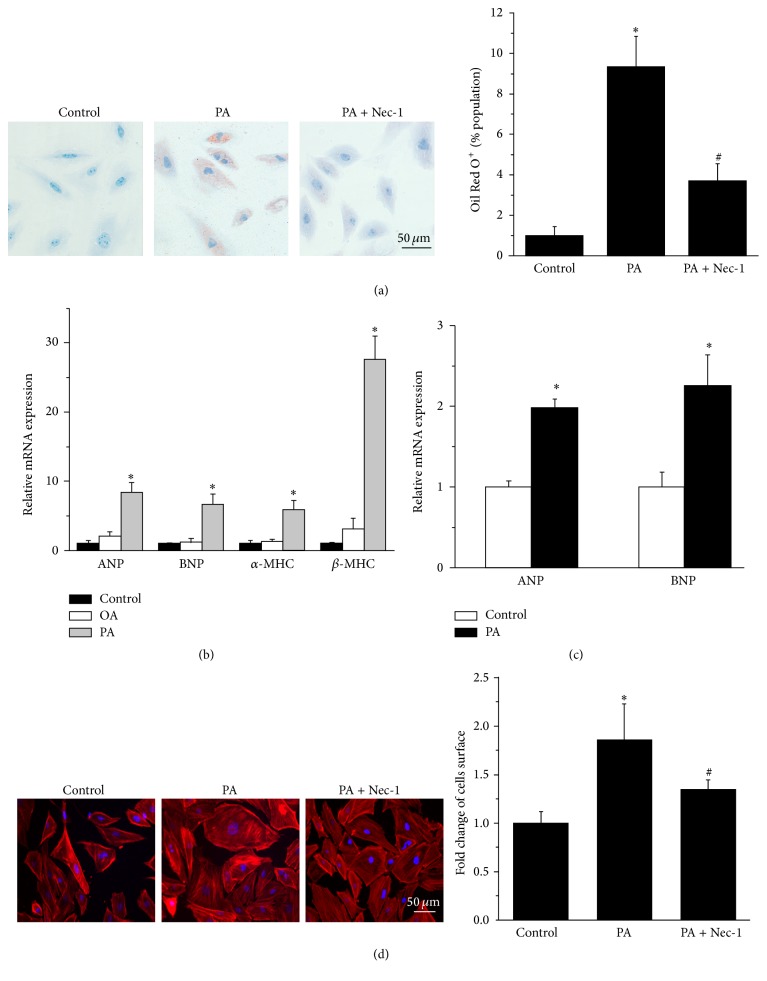
PA-induced hypertrophy in cardiomyocytes. (a) H9c2 cells were stained by Oil Red O dye. (1) Control (without any treatment). (2) Stimulation with PA (200 *μ*M). (3) Pretreatment with Nec-1 (10 nM), *n* = 3. The result of Oil Red O^+^ (%population) indicated that lipid accumulation was induced in PA stimulation group in H9c2 cells, and the PA-induced intracellular accumulation of neutral lipids in H9c2 cells decreased in PA + Nec-1 group. (b) Gene expression of ANP, BNP, *α*-MHC, and *β*-MHC in H9c2 cells was induced by PA, but not OA, *n* = 3. (c) Gene expression of ANP and BNP was upregulated in NCMs, *n* = 4. (d) Fluorescence microscopy observed the increased H9c2 cells surface area of F-actin staining in PA group, which was suppressed by Nec-1 (10 nM), according to the semiquantitative results by ImageJ software, *n* = 3. The red fluorescence indicated cytoskeleton stained by rhodamine phalloidin and the blue fluorescence indicated the cell nucleus stained by DAPI. Data in (a), (b), (c), and (d) are expressed as mean ± SD, *∗* indicates *p* < 0.05 compared to control treatment, and # indicates *p* < 0.05 compared to PA treatment.

In order to observe the lipotoxicity induced by PA administration in cardiomyocytes, we used Oil Red O, an agent that detects neutral lipids, to assess intracellular lipid accumulation in H9c2 cells under different treatments: (1) control (without any treatment); (2) stimulation with PA (200 *μ*M); and (3) pretreatment with Nec-1 (10 nM) and then exposure to PA. As shown in the representative images (Figure 1(a)), PA treatment induced lipids accumulation in H9c2 cells, and the PA-induced intracellular accumulation of neutral lipids in H9c2 cells could be inhibited by Nec-1 (Figure 1(a)).

Cardiomyocyte hypertrophy is one of consequences of lipotoxicity induced by PA treatment. We subsequently detected the changes in expression of hypertrophic marker genes after excessive PA supply in cardiomyocytes. Oleic acid (OA), the major unsaturated fatty acid in plasma which plays different pathophysiological roles [24], was set as another treatment measure for comparison. Thus, we treated H9c2 cells with PA (200 *μ*M) or OA (250 *μ*M) for 24 h. The results exhibited that gene expression of hypertrophic markers, atrial natriuretic peptide (ANP), brain natriuretic peptide (BNP), myocardial *α*-myosin heavy chain (*α*-MHC), and *β*-myosin heavy chain (*β*-MHC), was upregulated only in the PA stimulation group when compared with controls (without any treatment and with OA treatment) (Figure 1(b)). To confirm the prohypertrophy effect of PA, the primary rat NCMs were treated with PA (200 *μ*M) for 24 h and the gene expression levels of ANP and BNP were nearly 2-fold higher than those in the control (without any treatment) (Figure 1(c)).

We further measured the change of cell surface area by F-actin staining. The results illustrated that a cell surface area enlargement was observed in H9c2 cells treated with PA for 24 h (Figure 1(d)). The above data suggested that it is PA but not OA that could induce cardiomyocyte hypertrophy.

### 3.2. Necroptosis Is Induced in Cardiomyocytes with PA Stimulation and Is Involved in PA-Induced Cardiomyocyte Hypertrophy

**Figure 2:**
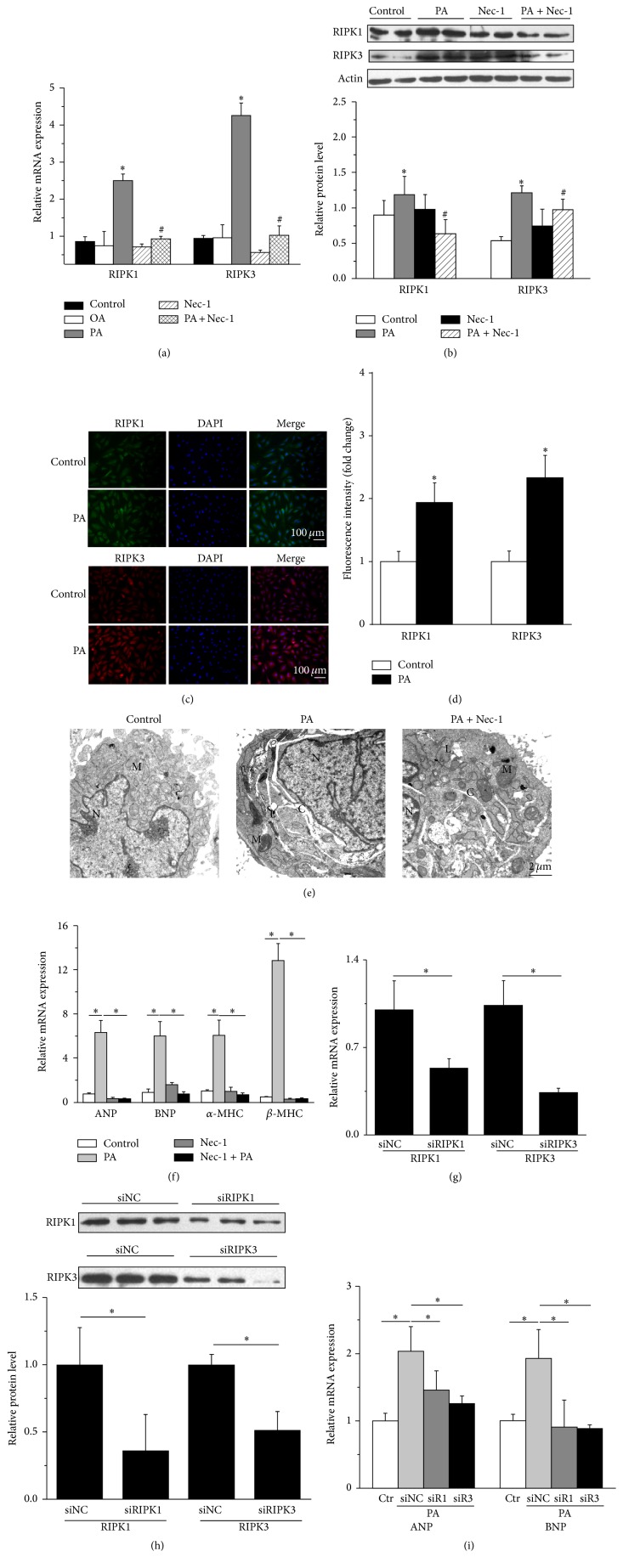
RIPK1/RIPK3 expressions are significantly increased in cardiomyocytes with PA stimulation. (a) The increased gene expression of RIPK1/RIPK3 was inhibited by Nec-1 (10 nM) in NCMs (*n* = 3). (b) The increased protein level of RIPK1/RIPK3 in NCMs was downregulated by Nec-1 (10 nM) (*n* = 4). (c) Immunofluorescence for RIPK1 and RIPK3 in H9c2 cells. Note the higher immunoreactivity for RIPK1/RIPK3 in H9c2 cells (200x) with PA stimulation. The green fluorescence indicated RIPK1 staining, the red fluorescence indicated RIPK3 staining, and the blue fluorescence indicated the cell nucleus stained by DAPI. (d) Quantitative analysis of fluorescent microscopy images (c) (*n* = 3). (e) Transmission electron microscopy images of H9c2 cells treated with PA for 24 h showed lipid deposition within the cells. Necrotic morphology was observed including swollen mitochondria, cytoplasmic clearing, and membrane damage (M: mitochondrion; N: nucleus; L: lipid droplet; C: cytoplasmic clearing. Scale bars: 2 *μ*m). (f) Treating the H9c2 cells with Nec-1 (10 nM), a specific necroptosis inhibitor, the increased gene levels of ANP, BNP, *α*-MHC, and *β*-MHC were downregulated via real-time PCR (*n* = 3). (g) After transfection with siRIPK1 or siRIPK3 (50 nM) in H9c2 cells, the PA-induced mRNA expression of RIPK1 or RIPK3 was significantly suppressed (*n* = 4 in each group). (h) After transfection with siRIPK1 or siRIPK3 (50 nM) in H9c2 cells, the PA-induced protein expression of RIPK1 or RIPK3 was significantly suppressed (*n* = 3). (i) Accordingly, silenced RIPK1 with siRIPK1 (siR1) or silenced RIPK3 (siR3) with siRIPK3 significantly inhibited both basal and PA-induced ANP and BNP gene expression in H9c2 cells (*n* = 3), as evaluated by quantitative RT-PCR. Data in (a), (b), (d), (f), (g), (h), and (i) are expressed as mean ± SD; *∗* indicates *p* < 0.05.

We investigated whether PA could induce necroptosis in cardiomyocytes. For this purpose, we first detected the gene expression of RIPK1 and RIPK3. After treatment with PA or OA for 24 h, the gene expression of both RIPK1 and RIPK3 in primary rat NCMs was upregulated only in the PA stimulation group compared with controls (without any treatment and with OA treatment), while Nec-1, a specific inhibitor of RIPK1, significantly decreased the PA-induced gene expression of RIPK1 and RIPK3 (Figure 2(a)).

Under the normal growth condition, the growth-arrested NCMs showed the low levels of RIPK1/RIPK3 protein expression. Western blot results showed that total cellular RIPK1/RIPK3 protein level was increased markedly after treatment with PA for 24 h and downregulated by Nec-1 (Figure 2(b)). The expression of RIPK1/RIPK3 protein obtained by immunofluorescence staining also indicated that growth-arrested H9c2 cells presented a slight RIPK1 and RIPK3 staining, while treatment with PA for 24 h significantly increased cytoplasmic RIPK1/RIPK3 staining (Figures 2(c) and 2(d)). We further found that expression of both RIPK1 and RIPK3 was repressed by Nec-1 pretreatment.

There are also ultrastructural features of necroptosis [25]. TEM analysis provided evidence for PA-induced lipid accumulation and alterations of ultrastructural features of necrosis, including swollen mitochondria, cytoplasmic clearing, cell membrane damage, and characteristic nuclear changes. Inhibition of RIPK1 with Nec-1 ameliorated necrotic characters of cardiomyocytes (reduction of cells fracture, etc.) (Figure 2(e)). The results displayed that PA stimulation could trigger necroptosis in cardiomyocytes.

Furthermore, we tested the hypothesis that RIPK1/RIPK3-mediated necroptosis may be actively involved in PA-induced cardiomyocyte hypertrophy. We observed that pretreatment with Nec-1 significantly decreased the mRNA expression of hypertrophy markers (including ANP, BNP, *α*-MHC, and *β*-MHC) (Figure 2(f)). Moreover, the result of F-actin staining illustrated that Nec-1 significantly decreased the augment of cell surface area induced by PA, as shown in Figure 1(d). Red Oil O staining also demonstrated that lipid accumulation in H9c2 cells induced by PA was attenuated by Nec-1 pretreatment, as shown in Figure 1(a).

To further investigate the role of necroptosis in PA-induced cardiomyocyte hypertrophy, we also used a gene silencing approach to specifically knockdown RIPK1 and RIPK3 expression. In order to confirm the inhibition effect of RIPK1/RIPK3-siRNA, we measured both the gene expression (Figure 2(g)) and the protein level (Figure 2(h)) of RIPK1 and RIPK3 in H9c2 cells after transfection. Subsequent to transfection of RIPK1-siRNA or RIPK3-siRNA into H9c2 cells, the PA-induced expression of RIPK1 or RIPK3 mRNA and protein were significantly suppressed. The knockdown of either RIPK1 or RIPK3 significantly reduced both basal and PA-induced ANP and BNP gene expression in H9c2 cells. As a negative control, the scrambled siRNA had no effect on ANP or BNP expression in H9c2 cells (Figure 2(i)). Altogether, cardiomyocyte hypertrophy induced by PA could be suppressed via specifically blocking RIPKs-dependent necroptosis.

### 3.3. The Endoplasmic Reticulum Stress Is Involved in the Palmitic Acid-Induced Necroptosis

**Figure 3:**
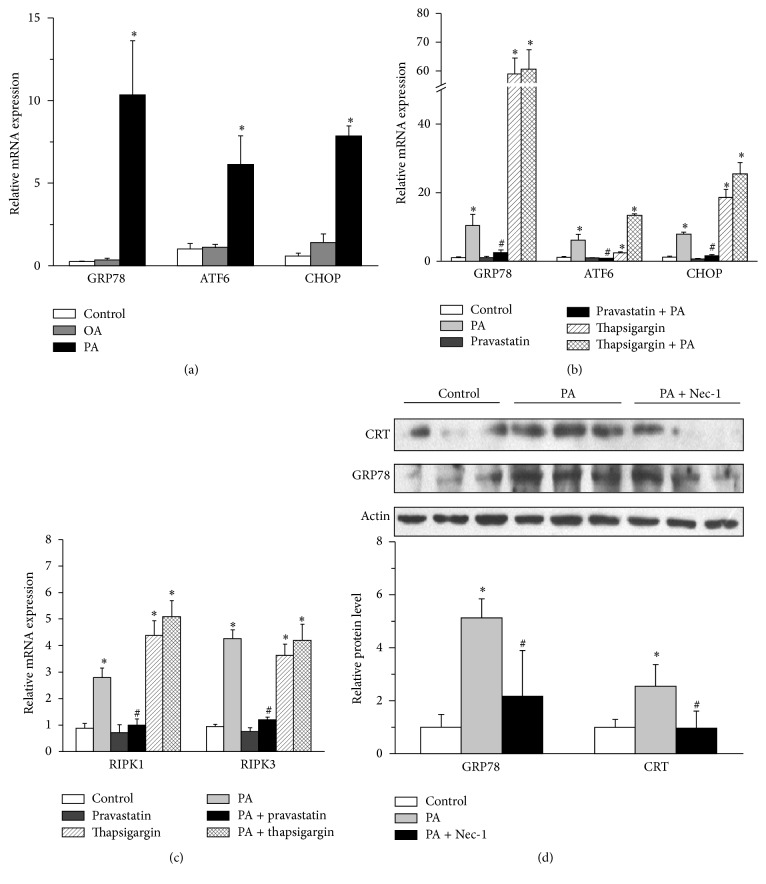
There is a crosstalk between ER stress and necroptosis in PA-induced cardiomyocyte hypertrophy. (a) Treating the NCMs with PA (200 *μ*M) and OA (250 *μ*M), the mRNA expressions of GRP78, ATF6, and CHOP were increased in PA group, not OA group, *n* = 4. (b) Pretreating the H9c2 cells with pravastatin (10 *μ*M) and effectively blocking the upregulated mRNA expression of GRP78, ATF6, and CHOP (ER stress markers) induced by PA and thapsigargin (100 nM) (an ER stress agonists) via real-time PCR, *n* = 3. (c) Pretreating the H9c2 cells with pravastatin (10 *μ*M) and also effectively blocking the upregulated mRNA expression of RIPK1/RIPK3 induced by PA and thapsigargin via real-time PCR, *n* = 3. (d) The increased protein level of GRP78 and CRT in NCMs was downregulated by pravastatin (10 *μ*M) (western blot), *n* = 3. Data in (a), (b), (c), and (d) are expressed as mean ± SD, *∗* indicates *p* < 0.05 compared to control treatment, and # indicates *p* < 0.05 compared to PA treatment.

We investigated whether there was a crosstalk between ER stress and necroptosis evoked by PA. In our experiments, we first treated the NCMs with PA (200 *μ*M) or OA (250 *μ*M) for 24 h. The gene expression of GRP78, ATF6, and CHOP, the markers of ER stress, was upregulated only in the PA stimulation group compared to OA treatment group (Figure 3(a)).

Then we pretreated H9c2 cells with pravastatin (10 *μ*M), which has been recognized as an inhibitor to ER stress. We also pretreated H9c2 cells with thapsigargin (100 nM), a known ER stress agonist. When the ER stress in H9c2 cells was suppressed, we observed that the ER stress markers were downregulated (Figure 3(b)) and the gene expression of RIPK1 and RIPK3 was also decreased compared to PA stimulation group (Figure 3(c)). Interestingly, when necroptosis was inhibited by Nec-1 in primary NCMs, the protein levels of the ER stress markers calreticulin (CRT) and GRP78 were also downregulated (Figure 3(d)).

### 3.4. mTOR Mediates the Necroptosis Induced Cardiomyocyte Hypertrophy

**Figure 4:**
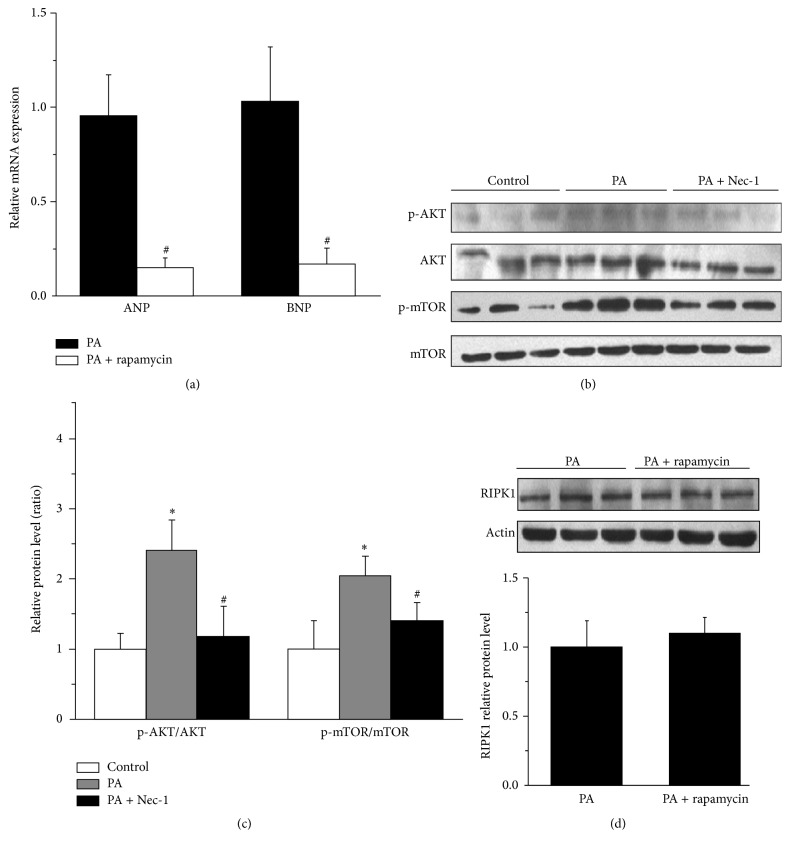
AKT/mTOR mediates necroptosis in the PA-induced cardiomyocyte hypertrophy. (a) Pretreating the NCMs with rapamycin (1 *μ*M), the mRNA level of ANP and BNP was decreased, *n* = 4. (b) Pretreating the NCMs with Nec-1 (10 nM), the upregulated Ser473 of AKT and Ser2481 of mTOR phosphorylation by PA stimulation (western blot) was inhibited. (c) Quantitative analysis of western blots (b), *n* = 3. (d) Pretreating the NCMs with rapamycin (1 *μ*M) had no effect on the upregulated RIPK1 by PA stimulation via western blot, *n* = 6. Data in (a), (c), and (d) are expressed as mean ± SD, *∗* indicates *p* < 0.05 compared to control treatment, and # indicates *p* < 0.05 compared to PA treatment.

It is recently reported that mTOR is involved in high-fat diet-Induced cardiac hypertrophy in mice [9], and AKT/mTOR mediates programmed necrosis in neurons [26]. Thus, we hypothesized that mTOR signaling participated in PA-induced necroptosis. Firstly, we observed the effect of rapamycin on cardiomyocyte hypertrophy with PA stimulation. Pretreating the NCMs with rapamycin could significantly decrease the mRNA level of ANP and BNP (Figure 4(a)), indicating that mTOR was involved in the PA-induced cardiac hypertrophy. To observe whether a crosstalk between necroptosis and AKT/mTOR signaling pathway exists, we pretreated NCMs with Nec-1 (10 nM) and rapamycin (1 *μ*M). After specifically blocking RIPK1 by Nec-1, the increased phosphorylation of AKT (Ser473) and mTOR (Ser2481) induced by PA stimulation was inhibited (Figures 4(b) and 4(c)), suggesting that activation of AKT/mTOR in response to PA in NCMs is RIPK1-dependent. However, pretreatment with rapamycin had no effect on the protein expression of RIPK1 (Figure 4(d)).

## 4. Discussion

In the present study, the potential link between PA-induced hypertrophy and necroptosis has been investigated. The major findings are that (1) necroptosis is involved in PA-induced cardiomyocyte hypertrophy; (2) there is a crosstalk between ER stress and necroptosis in PA stimulated hypertrophic cardiomyocytes; and (3) mTOR is identified as one of the molecular bases underlying PA-induced hypertrophy, which might be a downstream signaling molecule of RIPK1.

PA is the major saturated free fatty acid in plasma and is known to induce cellular dysfunction and cell death in a number of cell types, including cardiomyocytes [27]. Inflammation, hypertrophy, and cell death are major pathological events of the PA-induced cardiomyocyte lipotoxicity. The PA-induced inflammation and cell death including apoptosis and autophagy have been widely investigated in different cell types [24]. In the present study, therefore, we focus on the mechanism of the PA-induced cardiomyocyte hypertrophy. Several signaling pathways (e.g., LKB1/AMPK pathway) mediate the development of the PA-induced cardiac hypertrophy [28]. Consistent with previous reports, our data shows that exposure of primary rat NCMs or H9c2 cells to PA but not OA leads to an increase in expression of hypertrophy markers and a cell surface area enlargement. To further elucidate the underlying mechanism, we examined whether PA-induced cardiomyocyte hypertrophy was regulated by necroptosis, a novel cell death manner different from necrosis and apoptosis. It is negatively regulated by caspase and is dependent on the kinase activity of RIPK1 and RIPK3. We have detected an increased expression of RIPK1 and RIPK3 in PA-treated cardiomyocytes, implying activation of necroptosis [15, 22, 29, 30]. By using Nec-1 and specific siRNA for RIPK1 or RIPK3, our results demonstrate that inhibition of RIPK1 or knockdown of RIPK1/RIPK3 expression prevents the expression of hypertrophic marker genes effectively, indicating that necroptosis performs an important role in mediating cardiomyocyte hypertrophy in response to PA.

Necroptosis is regarded as a kind of cell death that is the caspase-independent programmed necrosis. It is involved in myocardial infarction and myocardial ischemia-reperfusion injury, whereas inhibition of both necroptosis and apoptosis could improve the cardioprotective effects [31]. In endothelial cells, the PA-induced necroptosis is carboxyl-terminal hydrolase- (CYLD-) dependent, but RIPK1- independent [32]. Nevertheless, we observed that the PA-induced cardiomyocyte hypertrophy is RIPKs-dependent. Nec-1 could inhibit RIPK1 expression, endogenous RIPK1 autophosphorylation, and even the formation of RIPK1-RIPK3 complex [22, 30, 33]. It has been used in many studies to test the contributions of RIPK1 and RIPK1-RIPK3 complex in cell death and inflammation [29, 33]. In our study, we observed that Nec-1 significantly reduced the intracellular lipid accumulation in PA-treated cardiomyocytes, suggesting that Nec-1 might protect cardiomyocyte from PA stimulation by repairing the cellular membrane damage. Moreover, Nec-1 pretreatment suppressed expression of both RIPK1 and RIPK3, demonstrating that the kinase complex which consisted of RIPK1 and RIPK3 might be essential in PA-induced cardiomyocyte necroptosis. Taken together, we have indicated that necroptosis is one of the main causes of the PA-induced cardiomyocyte hypertrophy.

It is interesting that by which approach the PA-induction of RIPKs-dependent necroptosis leads to cardiomyocyte hypertrophy. Some studies have shown that necroptosis mediates and even promotes the pathological processes of inflammation and cell death, and inhibition of this pathway can limit extensive tissue damage [13]. Elevated level of inflammation and apoptosis accounts mainly for the PA-induced lipotoxicity in cardiomyocytes [27, 34], consequently the inhibition of necroptosis attenuated cardiac hypertrophy. It is suggested that hypertrophy is accompanied by inflammation and apoptosis [35]. This might be the direct role of RIPK inhibition in mediating cardiac hypertrophy induced by PA.

In addition to the direct role, increased RIPKs expression may interact with other signals involved in cardiac hypertrophy. Several intracellular signals elicited by PA are responsible to cardiac hypertrophy, including ER stress and mTOR pathway. Previous studies have verified that ER stress is a pathological characteristic of cardiac hypertrophy, and the expression of related molecules such as GRP78, ATF6, CRT, and CHOP is increased significantly during cardiac hypertrophy [36]. An induction of the prolonged ER stress has also been proposed as a molecular mechanism of the PA-induced cardiomyocyte lipotoxicity [23]. Recently, it is reported that ER stress is able to induce necroptosis in L929 cells in a tumor necrosis factor receptor 1- (TNFR1-) dependent manner, but independent of autocrine TNF or lymphotoxin *α* production [37]. Therefore, we have investigated a possible crosstalk between ER stress and necroptosis in the PA-induced hypertrophic cardiomyocytes. Our study reveals that inhibiting of ER stress attenuated RIPKs expression induced by PA. On the other hand, blocking necroptosis decreased the expression of ER stress markers in cardiomyocytes. The effects of PA on ER stress may be different, depending on cell type and the duration of treatment. According to the present study, it is not sufficient to draw a conclusion that ER stress could evoke necroptosis in the PA-induced cardiomyocyte hypertrophy. However, there is a crosstalk between ER stress and necroptosis in mediating cardiomyocyte hypertrophy induced by PA, suggesting that necroptosis might play its role in the PA-induced cardiomyocyte hypertrophy via interaction with ER stress.

**Figure 5:**
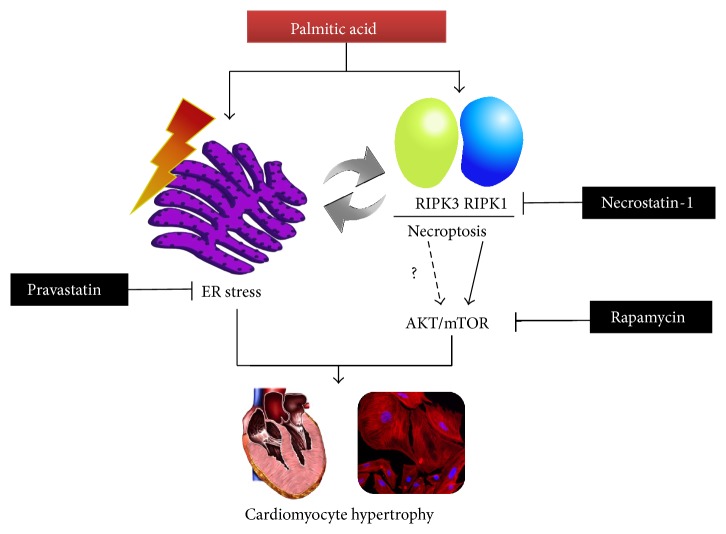
Schematic illustration of the signaling pathways of palmitic acid-induced cardiomyocyte hypertrophy.

Then we pay attention to AKT/mTOR pathway, which is activated in both physiological and pathological cardiac hypertrophy. Dysregulation of the mTOR pathway has been implicated in a number of human diseases such as obesity, diabetes mellitus, and cardiovascular diseases [38]. It has been reported that mTOR mediates RhoA-dependent [10] and hormone-induced cardiomyocyte hypertrophy [39], and previous report has shown that dual inhibition of AKT and mTOR reduced acute cell death [40]. AKT/mTOR mediates programmed necrosis in some types of cells such as RCC4 cells and neurons [26]. Recent study has shown that AKT is activated in a RIPK1-dependent way in L929 cells during necroptosis, and AKT regulates necroptosis via its downstream signaling complex mTORC1 [41]. These findings raise the possibility that mTOR mediates necroptosis in the PA-treated cardiomyocytes. We have observed that inhibition of necroptosis by Nec-1 attenuates the phosphorylation of AKT and mTOR induced by PA. However the mTOR inhibitor rapamycin has no influence on the expression of RIPK1, indicating that RIPK1 might be an upstream signal molecule of PA-induced AKT/mTOR activation. But, how about the effect of rapamycin on the activity of RIPK1 in PA-treated cardiomyocytes? To address this question, the phosphorylation of PIPK1 induced by PA may be needed to determine after rapamycin pretreatment in further study [42]. Here the current evidence suggests that necroptosis is involved in the PA-induced cardiac hypertrophy via activation of AKT/mTOR pathway. These phenomena summarized in Figure 5 indicate the processing of PA-induced cardiomyocyte hypertrophy.

In conclusion, necroptosis is involved in PA-induced cardiomyocyte hypertrophy and contributes to the pathogenesis of PA-induced lipotoxicity in the heart. Our work provides a novel insight into the mechanism of cardiac lipotoxicity and suggests a therapeutic potential of blocking necroptosis in the management of cardiac hypertrophy associated with elevated plasma concentration of FFAs.

## Figures and Tables

**Table 1:** Primer sequences and amplicon sizes for real-time RT-PCR.

Genes	GenBank ID	Primer sequence (5′-3′)	Amplicon (bp)
ANP	NM_012612.2	F: 5′ ACCAAGGGCTTCTTCCTCT 3′	141
		R: 5′ TTCTACCGGCATCTTCTCC 3′	

BNP	NM_031545.1	F: 5′ GCTCTTCTTTCCCCAGCTCT 3′	130
		R: 5′ ACTGTGGCAAGTTTGTGCTG 3′	

GRP78	NM_013083.2	F:5′ CCCCAGATTGAAGTCACCTTTGAG 3′	117
		R: 5′ CAGGCGGTTTTGGTCATTG 3′	

CHOP	NM_001109986.1	F: 5′ AGCAGAGGTCACAAGCACCT 3′	157
		R: 5′ CTCCTTCATGCGCTGTTTCC 3′	

ATF6	NM_001107196.1	F: 5′ GCAGGTGTATTACGCTTCGC 3′	136
		R: 5′ TGTGGTCTTGTTATGGGTGG 3′	

*α*-MHC	NM_017239.2	F: 5′ ATACCTCCGCAAGTCAGAGAA 3′	114
		R: 5′ ACGATCTTGGCCTTGACATAC 3′	

*β*-actin	NM_001099771	F: 5′ ACTATCGGCAATGAGCGGTTC 3′	77
		R: 5′ ATGCCACAGGATTCCATACCC 3′	

*β*-MHC	NM_017239.2	F: 5′ GTGCCAAGGGCCTGAATGAG 3′	353
		R: 5′ GCAAAGGCTCCAGGTCTGA 3′	

RIPK1	NM_001107350.1	F: 5′ CTTAAGCCCAAGTGCAGTCA 3′	166
		R: 5′ ATAGCCCAACAAGGAGGATG 3′	

RIPK3	NM_139342.1	F: 5′ CAGTGTTGGCTGGAAGAGAA 3′	173
		R: 5′ AGGCTCAGAACTCCAGCAAT 3′	
